# Drug Interaction-Informed Approaches to Inflammatory Bowel Disease Management

**DOI:** 10.3390/pharmaceutics16111431

**Published:** 2024-11-10

**Authors:** Kyeong-Ryoon Lee, Aneela Gulnaz, Yoon-Jee Chae

**Affiliations:** 1 Laboratory Animal Resource Center, Korea Research Institute of Bioscience and Biotechnology, Cheongju 28116, Republic of Korea; kyeongrlee@kribb.re.kr; 2 Department of Bioscience, University of Science and Technology, Daejeon 34113, Republic of Korea; 3 College of Pharmacy, Woosuk University, Wanju 55338, Republic of Korea; draneela@ws.ac.kr; 4 Research Institute of Pharmaceutical Sciences, Woosuk University, Wanju 55338, Republic of Korea

**Keywords:** inflammatory bowel disease (IBD), drug interactions, ulcerative colitis (UC), Crohn’s disease (CD)

## Abstract

Inflammatory bowel disease (IBD) is a complex and chronic condition that requires the use of various pharmacological agents for its management. Despite advancements in IBD research, the multifaceted mechanisms involved continue to pose significant challenges for strategic prevention. Therefore, it is crucial to prioritize safe and effective treatment strategies using the currently available pharmacological agents. Given that patients with IBD often require multiple medications due to combination therapy or other underlying conditions, a comprehensive understanding of drug interactions is essential for optimizing treatment regimens. In this review, we examined the pharmacological treatment options recommended in the current IBD management guidelines and provided a comprehensive analysis of the known pharmacokinetic interactions associated with these medications. In particular, this review includes recent research results for the impact of anti-drug antibodies (ADAs) on the concentrations of biological agents used in IBD treatment. By leveraging detailed interaction data and employing personalized dosing strategies, healthcare providers can improve therapeutic outcomes and minimize adverse effects, ultimately improving the quality of care for patients with IBD.

## 1. Introduction

Drug interactions occur when the pharmacological activity or clinical response to a drug is altered by the concomitant use of other substances, including drugs, foods, or dietary supplements [1]. These interactions can increase or diminish the drug efficacy or toxicity, potentially leading to therapeutic failure or unexpected side effects. Understanding the mechanisms of drug interactions is crucial for optimizing therapeutic effects and preventing adverse outcomes. Various underlying mechanisms contribute to drug interactions and can be categorized into two types: pharmacodynamic and pharmacokinetic interactions. Pharmacodynamic interactions occur when concomitant substances directly or indirectly affect receptor levels or share biochemical pathways, resulting in additive, synergistic, or antagonistic effects. Pharmacokinetic interactions involve changes in the absorption, distribution, metabolism, or elimination of drugs, altering their concentrations within the body, and thereby affecting their efficacy or toxicity [2]. Determining an appropriate drug dosage regimen based on accurate drug interaction information is essential for successful treatment outcomes.

Inflammatory bowel disease (IBD) is characterized by chronic and relapsing inflammatory disorders of the gastrointestinal tract. The prevalence of IBD is highest in North America and Europe, where 0.3–0.6% of the population suffers from IBD [3]. However, emerging data suggest that the prevalence of IBD is rapidly increasing in other regions, such as Asia, Africa, and South America [4]. IBD can develop at any age, but shows the highest incidence in those who are 15–30 years old and a smaller peak in those aged 50–70 years [5]. IBD is categorized into Crohn’s disease (CD) and ulcerative colitis (UC), depending on its pathophysiology. CD can affect any part of the gastrointestinal tract and involve the entire thickness of GI tract mucosa. In contrast, UC is limited to the colon, with continuous mucosal inflammation confined only to the mucosa and submucosa. Although understanding the contributing factors is crucial for the prevention of IBD, the exact etiology remains unclear and is likely to be multifactorial. Genetic factors are known to be associated with IBD and 240 risk variants for IBD have been reported by Genome-Wide Association studies (GWASs) to date, which include genes related with the intestinal epithelial barrier [e.g., nucleotide-binding oligomerization domain 2 (NOD2)] and genes encoding pro-inflammatory cytokines [e.g., tumor necrosis factor-α (TNF-α)] [6,7]. However, genetic variance accounts for only approximately 10–20% of IBD development [8]. Immunologically, IBD is characterized by an abnormal immune response where the body’s immune system attacks the intestinal mucosa, and thereby elevated levels of pro-inflammatory cytokines such al TNF-α, interleukin (IL)-1, and IL-6 are observed in patients with IBD. Environmental factors such as diet and smoking are also suggested as the main risk factors for IBD. Recent studies have highlighted the critical role of microbiota in IBD, showing reduced levels of beneficial bacteria such as *Bacteroides* and *Faecalibacterium prausnitzii* and increased levels of pro-inflammatory bacteria such as *Enterobacteriaceae* in patients with IBD [9,10,11].

**Figure 1:**
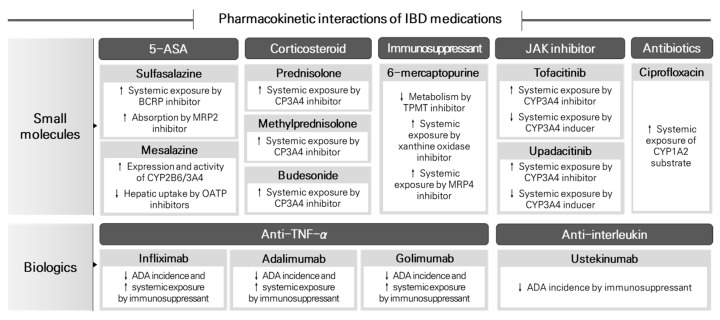
Drug interactions of medications used for IBD management.

Despite recent advancements in IBD research, multifaceted mechanisms present significant challenges for the strategic prevention of IBD. Thus, it is crucial to prioritize safe and effective treatment strategies using the currently available pharmacological agents. Given that patients with IBD frequently require multiple medications, owing to combination therapy or other underlying disease conditions, a comprehensive understanding of drug interactions is essential for optimizing treatment regimens. In this review, we examined the pharmacological treatment options recommended in the current IBD management guidelines and provided a comprehensive analysis of the known pharmacokinetic interactions associated with these medications (Figure 1). Notably, we included the latest research on the impact of anti-drug antibodies (ADAs) on the concentrations of biological drugs used in IBD treatment. The objective of this review was to facilitate a deeper comprehension of the interactions between drugs and their implications for the management of inflammatory bowel disease (IBD), thereby contributing to the advancement of more efficacious treatment strategies.

## 2. Mechanisms of Pharmacokinetic Interactions

The primary factors responsible for pharmacokinetic interactions with small molecules are drug-metabolizing enzymes and drug transporters. Drug-metabolizing enzymes are highly expressed in the liver, whereas other tissues, such as the intestine, lungs, and kidneys, express them to a lesser extent. Although many metabolizing enzymes have been identified, cytochrome P450 (CYP450) enzymes are the primary family responsible for the metabolism of several drugs [12]. CYP450 enzymes are a superfamily of heme-containing enzymes that metabolize approximately 75% of drugs. These enzymes can be inhibited competitively, noncompetitively, or uncompetitively, reducing the metabolism of substrate drugs and increasing their concentration in the body [13]. In contrast, the expression of metabolic enzymes can be increased by concomitant substances that act as ligands for nuclear receptors such as the pregnane X receptor (PXR), constitutive androstane receptor (CAR), and aryl hydrocarbon receptor (AhR) [14]. The increased expression of these enzymes enhances drug metabolism, leading to reduced drug exposure. Drug transporters, another key factor in drug interactions, are membrane proteins that play crucial roles in the movement of drugs across cellular membranes [15]. These are categorized as ATP-binding cassette (ABC) and solute carrier (SLC) transporters [16]. ABC transporters pump drugs out of cells using ATP. Multidrug resistance protein 1 (MDR1, also known as p-glycoprotein), a representative ABC transporter, is expressed in various tissues, such as the intestine and liver, where it hinders drug absorption and facilitates drug elimination. Breast cancer resistance protein (BCRP) and multidrug resistance-associated proteins (MRPs) are also ABC transporters. SLC transporters, including organic anion transporting polypeptides (OATPs), organic cation transporters (OCTs), and organic anion transporters (OATs), facilitate drug uptake into cells via electrochemical gradients. OATP1B1 and OATP1B3 are expressed in the liver and are involved in the hepatic uptake of drugs, such as statins and antiviral medications. OCT2, OAT1, and OAT3 are observed in the proximal tubule of the kidney, facilitating the movement of drugs from the bloodstream into the urine, thereby increasing the urinary excretion of drugs. In addition, other factors, such as alterations in gastrointestinal pH or mobility and the substitution of plasma protein binding, can induce drug interactions [17,18,19,20]. Pharmacogenetics should also be considered, as genetic variations can produce effects similar to drug interactions by altering the expression levels of metabolizing enzymes and transporters. Depending on the degree to which these enzymes and transporters influence drug pharmacokinetics, the impact of pharmacogenetic variations can vary significantly.

**Figure 2:**
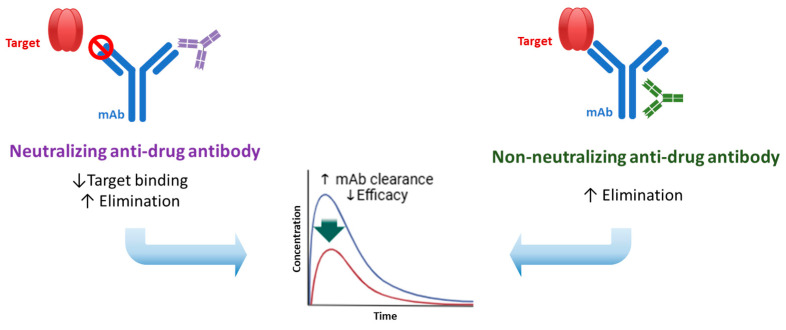
Impact of anti-drug antibody on pharmacokinetics of monoclonal antibody.

For large-molecule drugs, particularly monoclonal antibodies (mAbs), the frequency and mechanisms of pharmacokinetic interactions differ from those for small molecules. Owing to their large molecular size and specific target binding, pharmacokinetic interactions mediated by mAbs are less common than those mediated by small-molecule drugs. One crucial factor involved in the interaction of mAbs is the neonatal Fc receptor (FcRn) [21]. The FcRn protects IgG antibodies from lysosomal degradation, thereby extending their half-life in circulation [22]. Drugs that inhibit the FcRn may decrease mAb recycling, leading to faster clearance and reduced therapeutic efficacy. Conversely, enhancing FcRn function can prolong the half-life of mAbs. Another factor that contributes to drug interactions with mAbs is the presence of ADAs [23]. Neutralizing ADAs bind to mAbs and inhibit their interaction with the target antigen, thereby directly affecting their therapeutic effect. In addition, these complexes are rapidly eliminated from the body. Non-neutralizing ADAs do not block the binding of mAbs to the target but can still form immune complexes that are cleared more quickly. Both types of ADAs can consequently accelerate mAb clearance, although neutralizing ADAs have the added effect of reducing the pharmacodynamic activity of mAbs (Figure 2) [24]. The development of ADAs is influenced by several factors including the immunogenicity of the mAb, the patient’s immune system, and concomitant medications [25]. High immunogenicity increases the possibility of ADA formation, leading to more pronounced pharmacokinetic interactions. For example, drugs that stimulate the immune system may enhance ADA production, leading to increased monoclonal antibody clearance and reduced drug efficacy. Conversely, immunosuppressive drugs used in combination with mAbs can reduce the incidence of ADA formation, thereby increasing systemic exposure to mAbs.

As perpetrators, mAbs modulate CYP450 expression by altering cytokine levels. For example, mAbs that increase pro-inflammatory cytokine levels (e.g., IL-6) can decrease the expression of CYP450, thereby increasing systemic exposure to CYP substrates [26]. In contrast, mAbs that reduce cytokine levels (e.g., TNF-α inhibitors) may increase CYP expression and subsequently reduce the systemic exposure to CYP substrates.

## 3. Guidelines and Medications for Management of Inflammatory Bowel Disease

Various scientific societies and associations have published guidelines to support the optimal treatment of IBD. The American Gastroenterological Association (AGA) has issued three guidelines for the management of IBD: “Clinical Practice Guidelines on the Management of Mild to Moderate Ulcerative Colitis [27]”, “Clinical Practice Guidelines on the Management of Moderate to Severe Ulcerative Colitis [28]”, and “Clinical Practice Guidelines on the Medical Management of Moderate to Severe Luminal and Perianal Fistulizing Crohn’s Disease [29]”. The European Crohn’s and Colitis Organisation (ECCO) has published guidelines titled “Guidelines on Therapeutics in Ulcerative Colitis: Medical Treatment [30]” and “Guidelines on Therapeutics in Crohn’s Disease: Medical Treatment [31]”. The British Society of Gastroenterology (BSG) has issued a comprehensive guideline for IBD, named as “Guidelines on the Management of Inflammatory Bowel Disease in Adults [32]”. These guidelines aim to provide evidence-based recommendations for the management of IBD, thereby improving patient outcomes by standardizing care, reducing variability in treatment approaches, and ensuring the use of current and effective therapeutic strategies. Although the detailed contents of these guidelines differ according to specific healthcare systems, infrastructure, or special populations, pharmacological approaches for IBD management are similar among the guidelines.

In the management of mild to moderate UC, guidelines suggest the use of 5-aminosalicylic acid (5-ASA), such as sulfasalazine and mesalazine, as first-line therapy. If patients do not respond adequately to these treatments, corticosteroids such as prednisone and budesonide are recommended to induce remission, whereas immunomodulators such as azathioprine and 6-mercaptopurine (6-MP) are advised to maintain long-term remission. For moderate to severe UC, the guidelines suggest the use of biologics, including anti-TNF agents (e.g., infliximab, adalimumab, and golimumab), integrin inhibitors (e.g., vedolizumab), and IL-12/23 inhibitors (e.g., ustekinumab). Additionally, small-molecule drugs, such as Janus kinase (JAK) inhibitors (e.g., tofacitinib), are considered appropriate for these patients (Table 1).

For CD, the treatment approach for mild to moderate cases involves the use of corticosteroids such as prednisone and budesonide, especially for ileocecal diseases, and antibiotics such as metronidazole and ciprofloxacin are commonly used to manage perianal disease. In moderate to severe CD, the guidelines recommend corticosteroids to induce remission and the use of biologics, including anti-TNF agents (e.g., infliximab, adalimumab, and certolizumab pegol), integrin inhibitors (e.g., vedolizumab), and IL-12/23 inhibitors (e.g., ustekinumab) for both induction and maintenance therapy. Immunomodulators, such as azathioprine and 6-MP, are also included in maintenance strategies to help sustain remission (Table 1). For patients with severe or refractory CD who do not respond to standard medical therapies, biologics are a crucial component of treatment, and surgical interventions are considered when medical management fails to control the disease. 

This integrated approach ensures that treatment regimens are tailored to the severity and details of each patient’s condition, thereby optimizing outcomes and enhancing the overall quality of care for individuals with IBD. Given the need to customize therapies based on the specific conditions and characteristics of each patient with IBD, even small alterations in drug concentrations can significantly affect treatment outcomes. Especially for patients with severe IBD, combination therapy is often preferred over monotherapy. For instance, the AGA recommends using infliximab or adalimumab together with thiopurines or methotrexate to induce and maintain remission in those with moderate to severe CD, rather than relying on monotherapy [29]. Additionally, for adult outpatients with CD and active perianal fistula without a perianal abscess, the AGA recommends a combination of biologic agents with an antibiotic over a biologic agent alone to induce fistula remission. Consequently, a comprehensive understanding of drug interactions in IBD treatment is essential.

In addition, various natural products, though not officially approved by regulatory authorities for IBD treatment, are frequently used during IBD treatment. Approximately 10% of IBD patients have reported the use of herbal medicines [33]. Particularly, curcumin has shown potent anti-inflammatory effects in both in vitro and in vivo studies, and several clinical trials have evaluated its use as an add-on therapy for IBD [34]. Therefore, interactions between conventional IBD medications and natural products should also be closely investigated to ensure the effective and safe treatment of IBD.

## 4. Drug Interactions for the Drugs Used in IBD

### 4.1. 5-Aminosalicylates

5-ASAs are a class of anti-inflammatory drugs primarily used to treat IBD. Their therapeutic effects are primarily derived from the multifaceted mechanisms of action aimed at reducing inflammation in the gastrointestinal tract, although the precise mechanism of action is not entirely understood [35]. 5-ASAs inhibit the cyclooxygenase and lipoxygenase pathways, decreasing the production of pro-inflammatory prostaglandins and leukotrienes. 5-ASAs also modulate the nuclear factor kappa B (NF-κB) pathway, preventing the activation of genes involved in the inflammatory response [36]. They scavenge reactive oxygen species (ROS), reduce oxidative stress, and protect intestinal cells [37]. Additionally, 5-ASAs downregulate pro-inflammatory cytokines like TNF-α, IL-1β, and IL-6, further diminishing inflammation. Moreover, 5-ASAs enhance epithelial barrier function and promote mucosal healing by reducing epithelial cell apoptosis and increasing PPAR-γ expression [38].

Sulfasalazine, developed in the 1940s by Dr. Nanna Svartz, is a compound comprising sulfapyridine and mesalazine (5-aminosalicylic acid) linked by an azo bond. Initially intended to treat rheumatoid arthritis, it was later found to be effective for treating UC [39]. Sulfasalazine is poorly absorbed in the small intestine, partially due to efflux transporters such as BCRP and MRP2, and approximately 80–90% of the dose enters the colon, where bacterial azoreductases cleave the azo bond, releasing sulfapyridine and mesalazine [40]. Sulfapyridine is rapidly absorbed in the colon and metabolized in the liver via acetylation, hydroxylation, and glucuronidation. Mesalazine acts locally in the colon to exert anti-inflammatory effects and undergoes minimal systemic absorption [41]. When mesalazine is orally administered, it is prematurely absorbed or degraded in the upper gastrointestinal tract before reaching the colon, which is the target site. To ensure that mesalazine reaches its target site in the colon, various formulation strategies, such as enteric-coated tablets or delayed-release capsules, have been employed. These formulations protect the active compound as it transitions through the stomach and small intestine, ensuring its release into the colon, where the pH is higher [42]. Olsalazine, which consists of two 5-ASA molecules linked by an azo bond, and balsalazide, a prodrug in which 5-ASA is linked to an inert carrier molecule via an azo bond, are also used to maintain remission in UC [43].

Because sulfasalazine is a substrate of BCRP, its activity could affect the pharmacokinetics of sulfasalazine, especially during the absorption process (Table 2). After the oral administration of sulfasalazine, the area under the curve (AUC) in Bcrp1 knockout (KO) mice increased approximately 111-fold compared to that in wild-type (WT) mice, whereas intravenous administration of sulfasalazine increased the AUC in Bcrp1 KO mice by approximately 13-fold compared to that in WT mice, demonstrating the crucial role of BCRP in the absorption of sulfasalazine [44].

Curcumin, a potent bioactive compound, inhibits BCRP and affects the pharmacokinetics of sulfasalazine. Curcumin inhibited the transport of sulfasalazine in Caco-2 cells with an IC_50_ value of 17.4 μM [45] or in membrane vesicles expressing BCRP with a K_i_ value of 0.7 μM. In a study in which curcumin was administered to mice at 300 or 400 mg/kg, systemic exposure to sulfasalazine (10 mg/kg, PO) increased 8- to 8.5-fold, whereas no difference was observed in BCRP-knockout (KO) mice, highlighting the potent inhibitory effect of curcumin on BCRP, which enhances sulfasalazine absorption [46]. Similarly, when sulfasalazine (5 mg/kg) was administered orally to monkeys, systemic exposure increased after pretreatment with curcumin (30 mg/kg PO) [45]. Additionally, Kusuhara et al. [46] demonstrated the clinical significance of BCRP-mediated interactions with sulfasalazine, reporting a 3.2-fold increase in the AUC of sulfasalazine (2 g, PO) when curcumin (2 g, PO) was co-administered in humans. Quercetin, a naturally occurring flavonoid, has also been reported to inhibit BCRP; however, its in vivo inhibitory effects on BCRP remain controversial. The IC_50_ value of quercetin on sulfasalazine transport by BCRP was reported as 4.22 μM, and the pretreatment of quercetin (10 mg/kg, PO) into rats demonstrated a 1.8- and 1.5-fold increase in the AUC and the C_max_ of sulfasalazine (2 mg/kg, PO) in the study by Song et al. [47]. In contrast, Oh et el. [48] reported that the systemic exposure to sulfasalazine was not altered when sulfasalazine (20 mg/kg, PO) was co-administered with quercetin (100 mg/kg, PO) in rats. This discrepancy suggests that the inhibitory effects of quercetin on BCRP may depend on specific conditions, such as the dosage of sulfasalazine, warranting further investigation.

Another efflux transporter, MRP2, also plays a role in the pharmacokinetics of sulfasalazine. Indomethacin, an MRP2 inhibitor, decreased the efflux of sulfasalazine with an IC_50_ value of 75.1 μM in Caco-2 cells [51]. In addition, indomethacin increases the permeability of sulfasalazine in the rat jejunum, resulting in a shift in the Biopharmaceutics Classification System (BCS) class of sulfasalazine from IV to II.

These kinds of interactions, leading to the increased absorption of sulfasalazine, are likely to decrease the amount of sulfasalazine that reaches the colon, which is the target site. Therefore, concomitant medications that inhibit BCRP or MRP2 should be used with caution when administering sulfasalazine.

Mesalazine induces the expression and activity of CYP2B6 and 3A4 mRNA in human hepatocytes [52]. Whether mesalazine is produced by the breakdown of sulfasalazine or delivered directly to the colon via formulation, its absorption is likely to be minimal in the colon. Therefore, the clinical impact of CYP induction by mesalazine is likely to be limited; however, further research is needed to confirm this. Additionally, the potential to induce CYP450 enzyme expression in the intestine should be considered. Regarding drug interactions mediated by OATP transporters, mesalazine transport is mediated by OATP1B1, 1B3, and 2B1; therefore, co-administration with OATP inhibitors may alter the hepatic distribution or other pharmacokinetic parameters of mesalazine [53].

### 4.2. Corticosteroids

Corticosteroids achieve therapeutic efficacy in the management of IBD through a dual mechanism of action, encompassing both anti-inflammatory and immunosuppressive effects. These drugs primarily mimic the effects of endogenous glucocorticoids, which are hormones produced by the adrenal cortex that play a critical role in regulating inflammation and immune responses [54].

Prednisone, an oral corticosteroid commonly used for the treatment of IBD, is known for its effectiveness in quickly suppressing inflammation. After administration, it is metabolized by 11β-hydroxysteroid dehydrogenase type 1 (11β-HSD1) into prednisolone, an active metabolite that is also directly available as a medication and is frequently prescribed to IBD patients [55]. Both prednisone and prednisolone are well absorbed in the gastrointestinal tract, showing high bioavailability, ranging from 70–80% [56]. CYP3A4 is responsible for the metabolism of approximately 50% of prednisolone. The rest of the metabolism is managed by other enzymes, including 20α/β-hydroxysteroid dehydrogenase. These additional enzymes contribute to the overall metabolism of prednisolone, ensuring that the drug is effectively eliminated even when CYP3A4 activity is decreased by factors such as drug interactions. Although drug interactions with CYP3A4 inhibitors or inducers can affect prednisolone levels, the overall effect is generally moderate. For instance, multiple dosing of ritonavir, a strong inhibitor of CYP3A4 with an IC_50_ value of 0.014 μM [57] for 14 days, increased the AUC of prednisolone by 1.3-fold [58]. Diltiazem, a moderate inhibitor for CYP3A4 (IC_50_ 18 μM), increased the AUC of prednisolone by 1.2-fold, which is a marginal difference in systemic exposure [59] (Table 3).

Pichard et al. [70] reported that prednisone, but not prednisolone, induced CYP3A4 mRNA and protein expression in human hepatocytes at high concentrations (100 μM). However, prednisone did not significantly affect the pharmacokinetics of drugs extensively metabolized by CYP3A4, such as midazolam and odanacatib [60], in humans, indicating that it may not be sufficient to induce CYP3A4 in clinical settings, which alters the pharmacokinetics of these drugs.

Methylprednisolone, a synthetic corticosteroid with potent anti-inflammatory and immunosuppressive properties, undergoes hepatic oxidative metabolism, primarily via CYP450 enzymes, particularly CYP3A4. Co-administration of CYP3A4 inhibitors significantly altered the pharmacokinetics, leading to marked increases in plasma concentrations. For example, co-administration with itraconazole resulted in a 2.5–3.9-fold increase in the AUC of methylprednisolone [61,62,63], whereas co-administration with nefazodone led to a 2.2-fold increase [67]. These interactions led to prolonged cortisol suppression, extending the effect of methylprednisolone beyond 32 h compared to the typical 23.3 h under standard conditions. These findings underscore the importance of careful monitoring and possible dose adjustments when methylprednisolone is used in conjunction with CYP3A4 inhibitors to avoid enhanced systemic corticosteroid effects. Moreover, clinically significant drug interactions have been reported with other CYP3A4 inhibitors, such as aprepitant and grapefruit juice [64,66]. Therefore, caution is warranted when using these inhibitors concurrently with methylprednisolone.

Previous studies have explored the relationship between methylprednisolone and MDR1. Methylprednisolone is transported by MDR1, and its transport is inhibited by MDR1 inhibitors such as verapamil. However, the clinical significance of MDR1 remains unclear, and studies have shown contradictory results. For example, while some studies have found no significant difference in the methylprednisolone response among childhood idiopathic thrombocytopenic purpura patients with the MDR1 3435 C>T variant, others have reported differing responses in patients with rheumatoid arthritis depending on their MDR1 genotypes [71]. Further in vitro and large-scale cohort studies are required to improve our understanding of the role of MDR1 in the pharmacokinetics and therapeutic efficacy of methylprednisolone.

Budesonide is designed for localized action, particularly within the intestine, to minimize systemic side effects while effectively controlling inflammation. Budesonide undergoes extensive first-pass metabolism by CYP3A4, which results in a low systemic bioavailability of 10–20% following oral administration, effectively limiting its systemic effects and reducing the potential for side effects [72]. However, significant drug interactions have been reported, such as a 6.5-fold increase in systemic exposure when budesonide was co-administered with ketoconazole [68]. Grapefruit juice has also been shown to significantly increase the plasma concentrations of orally administered budesonide, likely owing to its inhibition of CYP3A4 during first-pass metabolism [69]. Although budesonide has also been reported as a substrate for MDR1, the impact of MDR1 inhibition on its absorption is expected to be minimal because of extensive CYP3A4 metabolism in the intestine [73]. Moreover, given the low systemic exposure and relatively high safety margins, the risk associated with increased exposure by MDR1 inhibitors was considered to be low. This assumption is further supported by clinical studies showing that MDR1 genetic variants do not significantly affect budesonide pharmacokinetics [74].

### 4.3. Immunosuppressants

Azathioprine and 6-MP are immunosuppressive drugs commonly used to manage IBD. Azathioprine is a prodrug of 6-MP that directly inhibits DNA and RNA synthesis and is crucial for immune cell division and function. These drugs help control the autoimmune processes that drive IBD by reducing immune cell activity [75]. However, given their potential for severe side effects, careful monitoring of systemic exposure is essential. One of the most critical toxicities of thiopurines is myelosuppression, which can result in leukopenia, thrombocytopenia, and anemia, as these drugs inhibit DNA synthesis, affecting rapidly dividing cells like those in bone marrow [76]. Thiopurines can also cause liver toxicity, leading to elevated liver enzymes, cholestasis, and, in rare cases, hepatic veno-occlusive disease [76]. Therefore, when using thiopurines for IBD treatment, it is crucial to carefully evaluate drug interactions to prevent serious fatal adverse events, with close attention to toxicity risks. Azathioprine is primarily metabolized to 6-MP through non-enzymatic processes, indicating that this conversion occurs spontaneously in the body, typically involving glutathione. Once 6-MP is formed, it is further metabolized via several pathways involving key enzymes. Thiopurine S-methyltransferase (TPMT) converts 6-MP to 6-methylmercaptopurine (6-MMP), which is further metabolized to 6-methylmercaptopurine ribonucleotides (6-MMPR). Hypoxanthine-guanine phosphoribosyltransferase (HGPRT) converts 6-MP to 6-thioinosine monophosphate (TIMP), which is subsequently converted into active 6-thioguanine nucleotides (TGNs). Xanthine oxidase (XO) metabolizes 6-MP to 6-thiouric acid [77]. The balance between these metabolic pathways is critical in determining the therapeutic efficacy and potential toxicity of azathioprine and 6-MP. For example, patients with low or no TPMT activity due to genetic variations accumulate high levels of TGNs, which significantly increase the risk of severe toxicity. It was reported that three variant alleles, TPMT*2, *3A, and *3C, are responsible for over 95% cases of lower enzyme activity [78]. Thus, the Clinical Pharmacogenetics Implementation Consortium (CPIC) recommends reducing the starting dose of azahioprune with an TPMT intermediate metabolizer (e.g., *1/*2, *1/*3A, *1/*3B, *1/*3C, *1/*4) and considering alternative nonthipurine immunosuppressant therapy with a TPMT poor metabolizer (*3A/*3A, *2/*3A, *3A/*3C, *3C/*4, *2/*3C, *3A/*4) [79], which highlights the importance of the TPMP enzyme activity level when using azathioprine.

Various TPMT inhibitors that decrease the metabolism of 6-MP have been identified. Nonsteroidal anti-inflammatory drugs (NSAIDs) such as mefenamic acid (K_i_ 39 μM), naproxen (K_i_ 52 μM), and tolfenamic acid (K_i_ 50 μM) have shown TPMT inhibition effects in human erythrocytes [80]. In addition, 5-ASA derivatives inhibited TPMT activity. For instance, sulfasalazine non-competitively inhibited human recombinant TPMT with an IC_50_ of 78 μM. In erythrocytes isolated from IBD patients, the IC_50_ of sulfasalazine ranged from 9.4 to 17.4 μM, depending on the TPMT activity levels. Olsalazine and its metabolite olsalazine-O-sulfate also exhibited TPMT inhibition with IC_50_ values of 23 μM and 70 μM, respectively. However, the clinical significance of 5-ASA in TPMT inhibition remains unclear. Achkar et al. [81] reported that in patients with CD receiving 6-MP therapy, the addition of sulfasalazine did not significantly influence 6-MP metabolism or Inflammatory Bowel Disease Questionnaire (IBD-Q) scores compared with 6-MP monotherapy. Similarly, Hande et al. [82] found that although 5-ASA therapy was associated with elevated 6-TGN levels in pediatric and adult patients taking 6-MP or azathioprine, this increase did not appear to be due to TPMT inhibition, as 5-ASA exposure did not alter 6-MMP levels. Moreover, recent research has indicated that concomitant 5-ASA treatment does not significantly affect 6-TGN levels in patients with IBD who are receiving thiopurines [83]. Nonetheless, reports of bone marrow suppression associated with the co-administration of 5-ASA highlight the need for further studies to elucidate the precise mechanisms underlying these interactions [84,85].

Xanthine oxidase, a key enzyme involved in the metabolism of 6-MP, plays a significant role in drug interactions and has important clinical implications. Studies have shown that pretreatment with allopurinol, a well-known xanthine oxidase inhibitor, led to a 3-fold increase in the AUC of orally administered 6-MP in monkeys [86]. In humans, this increase was even more pronounced, with AUC rising by 5-fold. In contrast, pretreatment with allopurinol did not affect the pharmacokinetics of intravenously administered 6-MP. This difference is likely due to the inhibition of the first-pass metabolism of orally administered 6-MP by xanthine oxidase in the liver and intestine. Interestingly, although allopurinol exhibited no inhibitory effect on TPMT, it has been reported that allopurinol increases exposure to 6-MP and 6-TGN, while decreasing the concentrations of 6-MMP and 6-MMPR [87]. Since 6-MMPR is associated with hepatotoxicity, co-administration of allopurinol reduced the risk of hepatotoxicity and doubled the maintenance period of 6-MP compared with those not receiving allopurinol [88]. Furthermore, in patients with IBD who demonstrate resistance to thiopurine therapy due to the preferential metabolism towards 6-MMP metabolites, the co-administration of allopurinol effectively improved disease activity and increased the percentage of patients achieving remission [89,90], suggesting an effective strategy for redirecting metabolic pathways towards the production of 6-TGN. Although these observations are presumed to be related to the metabolic pathways of 6-MP, the exact mechanisms have not yet been clearly identified. Further in-depth studies are needed to better understand and utilize these interactions in clinical practice.

Additionally, concurrent administration of methotrexate, a xanthine oxidase inhibitor, resulted in a 31% increase in the AUC and a 26% increase in the C_max_ of 6-MP, with the AUC of methotrexate correlating with the degree of increase in 6-MP plasma concentrations [91].

6-MP is a known substrate of MRP4 [92,93,94]. In studies using Mrp4 KO mice, there was a notable accumulation of 6-TGN in myelopoietic cells, which corresponds to increased toxicity [95]. Similarly, human patients carrying the MRP4 variant exhibited significantly higher levels of 6-TGN than those carrying the wild-type gene. This increase in 6-TGN levels was associated with a higher incidence of leukopenia in patients with MRP4 variants [96]. These findings suggest that MRP4 is one of the critical factors in the use of 6-MP, influencing not only the concentration of active metabolites but also the efficacy and toxicity of the drug. Therefore, MRP4-mediated drug interactions with 6-MP should be carefully considered in order to optimize therapeutic outcomes and minimize adverse effects.

### 4.4. JAK Inhibitors

The JAK-signal Transducer and Activator of Transcription (STAT) signaling pathway is critically involved in the transduction of extracellular cytokine signals, which regulate gene expression and drive various immune and inflammatory responses [97]. JAK inhibitors inhibit one or more JAK enzymes and interrupt subsequent signaling cascades, thereby preventing the phosphorylation and activation of STAT proteins. Consequently, JAK inhibitors attenuate cytokine-driven inflammatory processes.

**Table 4:** Pharmacokinetic interactions of tofacitinib.

Perpetrator	Victim	Experimental System	Interactions	Potential Mechanisms	Ref.
Baohuoside I	Tofacitinib	RLM	IC_50_ of 3.23 μM on tofacitinib metabolism	Cyp3A1/2 inhibition	[98]
		Rat	1.3- and 2.0-fold increase in AUC of tofacitinib (10 mg/kg, PO) by baohuoside I pretreatment (20 mg/kg, PO, single/7-day dosing)		
Bergapten	Tofacitinib	RLM/HLM/rhCYP3A4	IC_50_ of 2.6/2.6/1.4 μM on tofacitinib metabolism in RLM/HLM/rhCYP3A4	Cyp3A1/2 inhibition	[99]
		Rat	2.7- and 3.1-fold increase in AUC_inf_ of tofacitinib (10 m/kg, PO) by bergapten treatment (20 and 50 mg/kg, PO)		
Fluconazole	Tofacitinib	Healthy volunteers	1.8-fold increase in AUC_inf_ of tofacitinib (30 mg, PO) by fluconazole pretreatment (400 mg at day 1 and 200 mg at day 2–7, PO)	CYP3A4/2C19 inhibition	[100]
Isopsolaren	Tofacitinib	RLM/HLM/rhCYP3A4	IC_50_ of 19.3/12.3/17.1 μM on tofacitinib metabolism in RLM/HLM/rhCYP3A4	Cyp3A1/2 or CYP3A4 inhibition	[99]
		Rat	1.9- and 2.5-fold increase in AUC_inf_ of tofacitinib (10 mg/kg, PO) by isopsolaren treatment (20 and 50 mg/kg, PO)	Cyp3A1/2 inhibition	
Myricetin	Tofacitinib	RLM/HLM/rhCYP3A4	K_i_ of 6.5/7.5/1.9 μM on tofacitinib metabolism in RLM/HLM/rhCYP3A4	Cyp3A1/2 or CYP3A4 inhibition	[101]
Naringenin	Tofacitinib	Rat	1.8-fold increase in AUC_24h_ of tofacitinib (5 mg/kg, PO) by naringenin pretreatment (150 mg/kg, PO, 2 weeks)	Cyp3A1/2 inhibition	[102]
Ketoconazole	Tofacitinib	Rat	7.2-fold increase in AUC_inf_ of tofacitinib (10 mg/kg, PO) in the presence of ketoconazole (40 mg/kg, PO)	Cyp3A1/2 inhibition	[99]
		Healthy volunteers	2.0-fold increase in AUC of tofacitinib (30 mg, PO) Ketoconazole pretreatment (400 mg, PO, 3 days)	CYP3A4 inhibition	[100]
Resveratrol	Tofacitinib	RLM/HLM/rhCYP3A4	Ki of 19.2/11.6/5.4 μM on tofacitinib metabolism in RLM/HLM/rhCYP3A4	Cyp3A1/2 or CYP3A4 inhibition	[103]
		Rat	2.1- and 2.6-fold increase in AUC_inf_ and C_max_ of tofacitinib (1 mg/kg, PO) by resveratrol pretreatment (50 mg/kg, PO)	Cyp3A1/2 inhibition	
Voriconazole	Tofacitinib	RLM/RIM	Ki of 6.5/26.2 μM on tofacitinib metabolism in RLM/RIM	Cyp3A1/2 inhibition	[104]
		Rat	2.7-fold increase in AUC_inf_ of tofacitinib (10 mg/kg, IV) by voriconazole treatment (10 mg/kg, IV) 2.7- and 2.3-fold increase in AUC_inf_ and C_max_ of tofacitinib (20 mg/kg, PO) by voriconazole treatment (20 mg/kg, PO)		
Rifampin	Tofacitinib	Human	84 and 74% decrease in AUC and C_max_ of tofacitinib (30 mg, PO) by rifampin pretreatment (600 mg, PO, 7 days)	CYP3A4 induction	[105]
Tofacitinib	Ethinylestradiol, levonorgestrel	Healthy volunteers	No significant differences in systemic exposure to ethinylestradiol (30 μg) and levonorgestrel (150 μg) by tofacitinib pretreatment (30 mg, BID, PO, 11 days)	-	[106]
Tofacitinib	Midazolam	HLM	IC_50_ > 30 μM for CYP1A2, 2B6, 2C8, 2C9, 2C19, 2D6, and 3A4	CYP450 inhibition	[107]
		Human hepatocytes	CYP3A4 mRNA increased at 25 μM, but activity not increased CYP1A2 mRNA not increased	CYP450 induction	
		Healthy volunteers	No significant differences in systemic exposure to midazolam (2 mg, PO) by tofacitinib pretreatment (30 mg, BID, PO, 6 days)	-	
Tofacitinib	Voriconazole	Rat	No significant differences in systemic exposure to voriconazole (10 mg/kg IV or 20 mg/kg PO) by the treatment of tofacitinib (10 mg/kg IV or 20 mg/kg PO)	-	[104]

HLM, human liver microsome; rhCYP3A4, recombinant human CYP3A4; RIM, rat intestine microsome; RLM, recombinant liver microsome.

Tofacitinib is the first JAK inhibitor approved by the United States Food and Drug Administration (US FDA) and is used to treat moderate to severe UC. Following oral administration, tofacitinib is well absorbed, with an absolute bioavailability of approximately 74%. The drug undergoes extensive hepatic metabolism predominantly via CYP3A4 with a minor metabolic contribution from CYP2C19. These enzymes facilitate the conversion of tofacitinib into inactive metabolites [108]. Given the significant role of CYP3A4 in tofacitinib metabolism, CYP3A4 inhibitors may impede this metabolic process, thereby altering the pharmacokinetic profile of tofacitinib (Table 4). In vitro studies have confirmed that CYP3A4 inhibitors such as resveratrol and voriconazole inhibit the metabolism of tofacitinib [103,104]. In rats, the co-administration of ketoconazole (40 mg/kg, PO) led to a more than 7-fold increase in the AUC of tofacitinib [99], whereas resveratrol (50 mg/kg, PO) increased it by more than 2-fold [103]. In human studies, the co-administration of ketoconazole at clinically relevant doses (400 mg, PO for 3 days) resulted in a smaller, yet still significant, 2.1-fold increase in the AUC of tofacitinib [100]. Additionally, co-administration of fluconazole (400 mg on day 1 and 200 mg on day 2–7, PO), a potent CYP2C19 inhibitor and moderate CYP3A4 inhibitor, resulted in a 1.8-fold increase in the AUC of tofacitinib [100]. Based on findings indicating that CYP2C19 genetic variants have minimal impact on the pharmacokinetics of tofacitinib, the observed interaction with fluconazole is likely due to CYP3A4 inhibition rather than CYP2C19 inhibition [105].

The effects of CYP3A4 induction on tofacitinib pharmacokinetics have been reported in humans. Repeated dosing of rifampin (600 mg) resulted in the decrease in systemic exposure to tofacitinib (84 and 74% decrease in the AUC and C_max_, respectively) [105]. Therefore, the co-administration of tofacitinib with rifampin or other strong CYP3A inducers is not recommended to prevent the loss of efficacy.

Conversely, the role of tofacitinib as a perpetrator of drug interactions appeared to be minimal. Tofacitinib exhibited IC_50_ values exceeding 30 µM for major CYP isozymes, and while it did increase CYP3A4 mRNA levels at 25 µM, it did not enhance enzymatic activity [107]. Furthermore, clinical trials have demonstrated that tofacitinib does not significantly alter the systemic exposure to midazolam, a CYP3A4 probe substrate, nor does it cause significant changes in the plasma concentrations of the oral contraceptives ethinylestradiol and levonorgestrel [106].

Upadacitinib, a selective JAK1 inhibitor, was approved by the US FDA in 2019 for the treatment of moderate to severe UC. Similarly to tofacitinib, the bioavailability of upadacitinib is sufficiently high for oral administration, and it is extensively metabolized in the liver, predominantly by CYP3A4. Co-administration of ketoconazole increased the C_max_ and AUC of upadacitinib by 70% and 75%, respectively, whereas repeated dosing with rifampin decreased the C_max_ and AUC of upadacitinib by 50 and 60%, respectively, suggesting an important role of CYP3A4 in the pharmacokinetics of upadacitinib [109]. As a perpetrator, upadacitinib is a weak inhibitor for CYP2C9 and 3A4 (IC_50_ of 40.3 and 181–212 μM, respectively) and did not significantly alter the pharmacokinetics of ethinylestradiol and levonorgestrel in humans [110]. Upadacitinib increased the mRNA expression of CYP3A and CYP2B6 in a concentration-dependent manner and resulted in a minor increase in CYP1A2 mRNA in human hepatocytes, whereas no meaningful effect on exposure to bupropion (CYP2B6 substrate) was observed with multiple doses of upadacitinib in humans, suggesting negligible clinical implications of the CYP450 induction effects of upadacitinib [110,111].

### 4.5. Antibiotics

In patients with IBD, antibiotics are advised primarily for managing complications and infections rather than as a direct approach to reducing inflammation. Metronidazole and ciprofloxacin are frequently prescribed, particularly in cases of perianal CD with abscesses, fistulas, or other perianal issues. These antibiotics can be effective in controlling infections and minimizing inflammation in the affected regions [112].

Ciprofloxacin is a broad-spectrum antibiotic that belongs to the fluoroquinolone class. It is rapidly absorbed after oral administration, with a bioavailability of about 70–80%. It is primarily excreted by the kidneys, with about 40–50% excreted unchanged in the urine. The remaining portion is eliminated as a metabolite generated primarily by CYP1A2 in the liver, or through bile into the gastrointestinal tract [113]. The inhibitory potential of ciprofloxacin on CYP1A2 is known to be weak (IC_50_ of 135 [114] or 220 μM [115]); however, its clinical implications of drug interactions are unexpectedly significant for some victim drugs. For example, orally administered ciprofloxacin (500 mg, TID for 3 days) significantly increased the AUC_inf_ of tizanidine (4 mg, PO), which is mainly metabolized by CYP1A2, by 10-fold and the C_max_ by 7-fold, resulting in stronger effects on blood pressure than the control group [116]. Relatively weak interaction effects were observed when clozapine was co-administered with ciprofloxacin, with a 29% increase in the mean serum concentration of clozapine [117]. Although some in vitro observations of drug interactions mediated by drug transporters, such as OAT1, OAT3, and OATP1A2, have been reported for ciprofloxacin [118,119], additional supporting data on these interactions are necessary to determine their clinical significance.

Interestingly, when ciprofloxacin was co-administered with sildenafil, which is predominantly metabolized by CYP3A4, the AUC of sildenafil increased by more than 2-fold, even though ciprofloxacin is not a known inhibitor of CYP3A4 [120]. Further investigations into the underlying mechanisms of this interaction are necessary for the safer and more effective use of ciprofloxacin.

Metronidazole is an antibiotic commonly used to treat various infections caused by anaerobic bacteria and certain protozoa by inhibiting nucleic acid synthesis in microorganisms. Metronidazole is metabolized via various pathways, including side-chain hydroxylation, oxidation, and glucuronidation [121]. Although CYP3A4 is partially involved in metronidazole metabolism, no significant clinical interactions were observed when metronidazole was co-administered with CYP3A4 inhibitors. Additionally, several studies have indicated that metronidazole has low potential to inhibit CYP3A4 in humans [122,123]. Therefore, based on current information, interactions mediated by CYP3A4 do not generally pose a significant concern with metronidazole.

### 4.6. Biologics

The introduction of mAbs has significantly altered the therapeutic landscape of IBD, providing targeted, biologically driven treatment options that have improved patient outcomes. The mAbs used in IBD treatment are designed to specifically target key molecules involved in disease pathogenesis, modulate immune responses, and reduce inflammation. The primary targets of mAbs in IBD treatment include tumor necrosis factor-alpha (TNF-α), integrins, and interleukins [124].

Anti-TNF-α agents, such as infliximab, adalimumab, and certolizumab pegol, bind to TNF-α, thereby preventing it from interacting with its receptors on the cell surface. The inhibition of TNF-α reduces the recruitment and activation of inflammatory cells, decreases the production of other pro-inflammatory cytokines, and ultimately lowers inflammation in the gut mucosa [125]. Infliximab was the first mAb approved for both CD and UC. Intravenously administered infliximab exhibited a biphasic distribution pattern. Its half-life ranges from 7 to 19 days depending on various patient-specific factors, including the presence of anti-drug antibodies (ADAs), the disease state, and other factors [126]. The development of ADAs significantly influences the pharmacokinetics of infliximab. For instance, in patients with ADAs, the clearance rate was found to be 0.768 L/day compared to 0.288 L/day in those without ADAs, highlighting the critical impact of ADAs on infliximab pharmacokinetics [127]. Clinical trials in patients with IBD have shown that the concurrent use of immunosuppressants such as azathioprine, 6-MP, and methotrexate increases the serum concentration of infliximab by decreasing the incidence of ADAs. (Table 5). For instance, Vermeire et al. [128] reported that the incidence of ADAs was 73% in patients receiving infliximab alone, whereas this rate dropped to 44–48% when immunosuppressants (i.e., azathioprine, 6-MP, or methotrexate) were used in combination with infliximab. Correspondingly, the infliximab concentrations were observed to be 2.42 and 6.45 μg/mL, respectively. In addition, Polakovicova et al. [129] found that higher doses of azathioprine were associated with increased systemic exposure to infliximab (2.83, 4.91, 5.67, and 7.53 μg/mL for azathioprine doses of <1 mg/kg, 1–2 mg/kg, and >2 mg/kg, respectively). The incidence of ADAs is also correlated with clinical outcomes. In a study by Colombel et al., the rate of steroid-free remission was 66.7–70.3% in ADA-negative patients, compared to 56.3–57.1% in ADA-positive patients [130]. Therefore, immunosuppressants may play a beneficial role in maintaining the efficacy of infliximab, and their use could be considered when there is concern regarding the reduced effectiveness of infliximab due to the presence of ADAs.

Adalimumab, another TNF-α inhibitor, is administered subcutaneously, greatly enhancing convenience for patients. The absolute bioavailability after subcutaneous injection was approximately 64%, with a time to reach C_max_ of 5.5 days. Similarly to infliximab, various studies have indicated that the presence of ADA is associated with increased drug clearance, which reduces its effectiveness. For instance, in patients with axial spondyloarthritis, ADAs were detected in 25% of patients receiving concomitant methotrexate, compared with 47.3% in those treated with adalimumab alone (*p* = 0.03). Moreover, the adalimumab concentrations were significantly higher in the group taking methotrexate at all time points [131]. As the methotrexate dose increased to 2.5, 5, 10, and 20 mg, the adalimumab trough concentrations correspondingly rose to 4.4, 5.7, 6.5, and 6.9 μg/mL at Week 26 in rheumatoid arthritis patients [132]. However, direct evidence demonstrating that immunosuppressants such as methotrexate affect ADA formation and alter adalimumab concentrations in patients with IBD remains unclear. The effect of immunosuppressants may vary depending on the disease context, highlighting the urgent need for more focused research to understand the context-specific effects of immunosuppressants in patients with IBD.

MTX is believed to suppress the immune system, thereby reducing ADA formation. However, recent studies in rats have suggested a different mechanism. Methotrexate may increase the expression of the FcRn, which enhances the recycling of adalimumab, leading to higher drug concentrations [133]. Specifically, FcRn concentrations in methotrexate-treated rats were significantly higher in both the liver and kidneys than in placebo-treated rats. The clearance (CL/F) of adalimumab was lower in the methotrexate-treated rats than in the placebo-treated rats, indicating slower drug elimination. The potential role of the FcRn in this process, as suggested by recent animal studies, warrants further investigation to clarify its role in adalimumab pharmacokinetics.

**Table 5:** Associations of mAb levels with immunosuppressant comedications in IBD patients.

mAb	Immunosuppressant	Subject	Outcomes			Ref.
Infliximab	AZA	Moderate to severe CD who had not undergone previous immunosuppressive or biologic therapy	Group	IFX trough levels at W30/46	Patient with steroid-free remission (%) at W30/50	[130]
			Infliximab	1.6/1.0 μg/mL	ADA negative: 66.7/70.6% ADA positive: 56.3/57.1%	
			Infliximab + AZA (2.5 mg/kg, QD)	3.5/3.8 μg/mL		
Infliximab	AZA	IBD patients in clinical remission	Group	Infliximab trough levels	Patient% with subtherapeutic levels of infliximab (3 μg/mL)	[129]
			Infliximab	2.83 μg/mL	57%	
			Infliximab + AZA (<1 mg/kg)	4.91 μg/mL	26%	
			Infliximab + Aza (1–2 mg/kg)	5.67 μg/mL	25%	
			Infliximab + AZA (>2 mg/kg)	7.53 μg/mL	11%	
Infliximab	AZA	IBD patients with infliximab maintenance therapy	Group	Patient with ADA (%)		[134]
			6-TGN level between 235 and 450 pmol/8 × 10^8^ RBC	18.8%		
			6-TGN level < 235-pmol/8 × 10^8^ RBC	63.6%		
Infliximab	MTX	CD patients who had initiated prednisone induction therapy within the preceding 6 weeks	Group	Infliximab level	Patient with ADA (%)	[135]
			Infliximab	3.75 μg/mL	20%	
			Infliximab + MTX (10 mg QW, escalating to 25 mg QW)	6.35 μg/mL (*p* = 0.08)	4%	
Infliximab	AZA, 6-MP, or MTX	CD patients treated with infliximab in an on-demand schedule	Group	Infliximab level	Patient with ADA (%)	[128]
			Infliximab	2.42 μg/mL	73%	
			Infliximab + AZA (2–2.5 mg/kg), 6-MP (1–1.25 mg/kg) or MTX (15 mg QW after induction for 12 W at 25 mg)	6.45 μg/mL	AZA or 6-MP: 48% MTX: 44%	
Infliximab	AZA,6-MP, MTX	CD patients starting infliximab treatment	Group	Infliximab level		[136]
			Infliximab	>12 μg/mL		
			Infliximab + AZA (2–2.5 mg/kg/day), 6-MP (1–1.25 mg/kg/day) or MTX (15 mg, QW)	<5 μg/mL		
Infliximab or adalimumab	MTX	Pediatric CD patients initiating infliximab or adalimumab	Group	Patient with ADA (%)		[137]
			Infliximab/ adalimumab	47/21%		
			Infliximab/ adalimumab + MTX (10–15 mg depending on body weight, QW)	34/15% (not statistically significant)		

6-MP, 6-mercaptopurine; 6-TGN, 6-thioguanine nucleotides; ADA, anti-drug antibody; AZA, azathioprine; IFX, infliximab; MTX, methotrexate.

Although there are no direct studies on the effect of methotrexate on golimumab concentrations in patients with IBD, research on psoriatic and rheumatoid arthritis has indicated that methotrexate can significantly influence the clearance of golimumab. In patients with psoriatic arthritis, methotrexate reduced the clearance of golimumab by approximately 10% [138], whereas in patients with rheumatoid arthritis, the reduction was much more significant (65%) [139]. This suggests that the effect of methotrexate on the pharmacokinetics of golimumab varies depending on the underlying conditions. Although the exact underlying mechanism on reduction in clearance has not been directly confirmed in humans, a recent study in monkeys demonstrated that methotrexate delayed the onset and reduced the magnitude of ADA formation, which correlated with reduced golimumab clearance [140].

Vedolizumab selectively blocks the interaction between α4β7 integrin and mucosal addressin cell adhesion molecule-1, primarily expressed in the gut. This interaction is crucial for the migration of lymphocytes into the gastrointestinal tract where they can cause inflammation and lead to UC and CD symptoms [141]. Population pharmacokinetic–pharmacodynamic modeling has shown that immunosuppressants such as thiopurines or methotrexate do not significantly affect the clearance of vedolizumab [142]. Moreover, although inflammatory cytokines can reduce CYP450 expression and anti-inflammatory treatments may increase its activity, vedolizumab does not appear to affect CYP450 enzyme activity. Specifically, clinical trial data demonstrated that there was no significant change in the 4β-hydroxycholesterol to cholesterol (4β-OHC/C) ratios—a marker for CYP3A4 activity—in patients with UC or CD before and during vedolizumab treatment [143]. This finding suggests that vedolizumab does not modulate CYP3A4 activity, implying a lack of significant drug–drug interactions involving this enzymatic pathway.

Ustekinumab is a mAb that targets the p40 subunit shared by the cytokines IL-12 and IL-23. By binding to this subunit, ustekinumab inhibits cytokines, which play crucial roles in the differentiation and activation of Th cells (Th1 and Th17). These Th cells are involved in inflammatory processes that contribute to autoimmune diseases such as UC and CD. By blocking IL-12 and IL-23, ustekinumab effectively reduces the inflammatory response and alleviates the symptoms associated with these conditions [144]. Since ustekinumab is a fully humanized monoclonal antibody, in contrast to infliximab, which is a chimeric monoclonal antibody, the incidence of ADAs with ustekinumab is relatively low, with reports indicating an incidence of less than 5% in patients with CD [145]. Although ADA incidence is low, median serum ustekinumab concentrations were approximately two to three times lower in ADA-positive subjects compared to ADA-negative subjects. Additionally, the concomitant use of immunomodulators such as 6-MP, azathioprine, and methotrexate impacted immunogenicity; ADA positivity was observed in a lower proportion of subjects who received immunomodulators (1.9%) compared to those who did not (2.6%) [146]. However, the use of these immunomodulators did not significantly affect ustekinumab concentrations, based on serum concentrations and population pharmacokinetic analysis [147]. These contrary results may be due to the low ADA formation rate with ustekinumab, which limits the number of ADA-positive patients available for comparison. Further investigation with a larger cohort would help clarify the effects of immunosuppressants on ustekinumab pharmacokinetics.

## 5. Perspective

IBD is a complex and chronic condition that requires the use of various pharmacological agents. These therapeutic regimens often include combinations of anti-inflammatory agents, immunosuppressants, biological agents, and other supportive medications. In addition to the primary treatment options for IBD, patients frequently require medications to manage comorbid conditions such as cardiovascular diseases, osteoporosis, or infections, which further complicate their pharmacotherapy. Given this complexity, careful evaluation of potential drug interactions is essential to avoid adverse effects and therapeutic failure.

Among the drugs commonly used in IBD management, 5-ASA, corticosteroids, and immunosuppressants have been mainstays of therapy for decades. Despite their widespread use, there is a substantial lack of comprehensive data regarding their pharmacokinetic interactions when used in combination with other therapeutic agents. This knowledge gap can lead to either the underestimation or overestimation of drug interactions, potentially resulting in suboptimal dosing strategies or an increased risk of adverse effects. Further research is urgently needed to bridge these gaps and enhance our understanding of the interactions between these drugs.

Furthermore, it is crucial to recognize that the nature and extent of drug interactions may vary depending on the specific subtype of IBD (e.g., CD vs. US) and an individual patient’s disease state. Variations in inflammatory burden, mucosal integrity, and other pathophysiological factors can influence drug absorption, distribution, metabolism, and excretion. As a result, the direct application of interaction data from studies conducted in other disease conditions or even in healthy populations may not be appropriate for patients with IBD. This necessitates the development of IBD-specific interaction studies and the creation of tailored guidelines for drug use in this unique population.

Biological therapies such as TNF-α inhibitors, integrin inhibitors, and IL-12/23 inhibitors have further broadened the landscape of IBD treatment. These agents, although highly effective, present additional challenges in terms of their potential interaction with traditional therapies and other medications. The immunomodulatory effects of biologics can alter the pharmacokinetics of concomitantly administered drugs, necessitating careful consideration of the timing, dosing, and monitoring. In this context, the integration of advanced pharmacometric modeling and real-world evidence into clinical practice has become increasingly important. Further applications of pharmacokinetic–pharmacodynamic modeling will help predict potential interactions and guide dose adjustments.

## 6. Conclusions

The management of patients with IBD requires a comprehensive and nuanced understanding of drug–drug interactions. Clinicians must be vigilant when assessing potential interactions and adjusting treatment protocols accordingly. Continuous research is critical to fill the existing knowledge gaps, particularly concerning the pharmacokinetics of both traditional and emerging therapies for IBD. By leveraging detailed interaction data and employing personalized dosing strategies, healthcare providers can improve therapeutic outcomes and minimize adverse effects, ultimately improving the quality of care for patients with IBD.

## Figures and Tables

**Table 1:** Current guideline-based medications used in the management of IBD.

Category	Classification	Ulcerative Colitis (UC) [27,28,30,32]	Crohn’s Disease (CD) [29,31,32]	Medications
Small molecule	5-aminosalicylate (5-ASA)	Induction and maintenance of remission for mild to moderate UC	-	Sulfasalazine, mesalazine, olsalazine, balsalazide
	Corticosteroids	Induction of remission in moderate to severe UC when 5-ASA fails to induce remission	Induction of remission in moderate to severe CD	Prednisolone, hydrocortisone, budesonide, prednisone, methylprednisolone
	Immunosuppressant	Maintenance of remission in steroid-dependent moderate to severe UC patients	Maintenance of remission in moderate to severe CD	Azathioprine, 6-mercaptopurine
	JAK inhibitor	Induction and maintenance of remission in patients with moderate to severe UC who have inadequate response or intolerance to conventional therapy	-	Tofacitinib
	Antibiotics	-	Management of complications such as abscesses and fistulas	Metronidazole, ciprofloxacin
Monoclonal antibody	Anti-TNF-α	Induction and maintenance of remission in moderate to severe UC	Induction and maintenance of remission in moderate to severe CD	Infliximab, adalimumab, golimumab, certolizumab pegol
	Anti-integrin	Induction and maintenance of remission with moderate to severe UC, particularly useful for patients who do not respond to TNF-α antagonists	Induction and maintenance of remission in patients who do not respond adequately to TNF-α antagonists	Vedolizumab
	Anti-interleukin	Induction and maintenance of remission in moderate to severe UC when other biologics are ineffective or not tolerated	Induction and maintenance of remission in moderate to severe CD when other biologics are ineffective or not tolerated	Ustekinumab

**Table 2:** BCRP-mediated interactions with sulfasalazine as a victim drug.

Perpetrator	Experimental System	Interactions	Ref.
Curcumin	Caco-2	IC_50_ 17.4 μM for the transport of sulfasalazine	[45]
	Membrane vesicle	K_i_ 0.70 μM for the transport of sulfasalazine by membrane vesicle expressing hBCRP	[46]
	Mouse	8.5- and 8.0-fold increase in AUC_8h_ of sulfasalazine (10 mg/kg, PO) by curcumin pretreatment (300 or 400 mg/kg, PO) in WT mice No significance differences by curcumin pretreatment (400 mg/kg, PO) in Bcrp KO mice	[46]
	Monkey	2.9- and 4.1-fold increases in AUC_last_ and C_max_ of sulfasalazine (5 mg/kg, PO) by curcumin pretreatment (30 mg/kg, PO) No significant alteration in PK of sulfasalazine (5 mg/kg, IV) by curcumin pretreatment (30 mg/kg, PO)	[45]
	Healthy volunteers	2.0- and 3.2-fold increase in AUC_24h_ of sulfasalazine (100 μg and 2 g, PO) by curcumin pretreatment (2 g, PO)	[46]
Quercetin	Rat	1.8- and 1.5-fold increase in AUC_8h_ and C_max_ of sulfasalazine (2 mg/kg, PO) by quercetin pretreatment (10 mg/kg, PO)	[47]
	Rat	No significant alterations in PK of sulfasalazine (20 mg/kg, PO) by quercetin treatment (100 mg/kg, PO, single dosing or multiple dosing for 7 days)	[48]
	Beagle dog	No significant alterations in PK of sulfasalazine (50 mg/kg, PO) by quercetin treatment (1000 mg/head, PO, single dosing or multiple dosing for 7 days)	[48]
Gefitinib	Mouse	13-fold increase in AUC of sulfasalazine (20 mg/kg, PO) by gefitinib pretreatment (50 mg/kg, PO)	[44]
Pantoprazole	Caco-2	Decrease in efflux ratios of sulfasalazine by 60 and 81% in the presence of pantoprazole (20 and 100 μM)	[49]
Rolapitant	Healthy volunteers	2.3-fold and 2.4-fold increase in AUC_last_ and C_max_ and of sulfasalazine (500 mg, PO) by rolapitant treatment (180 mg, PO) No significant alterations in PK of sulfasalazine (500 mg, PO) by rolapitant treatment (166.5 mg, IV)	[50]

**Table 3:** CYP3A4-mediated clinical drug interactions for corticosteroids.

Potential Mechanisms	Study Design			Alterations of Systemic Exposure to Victim Drugs	Ref.
	Perpetrator	Victim	Subjects		
CYP3A4 induction	Prednisone (10 mg, PO, 2 or 4 weeks)	Midazolam (2 mg, PO), odanacatib (50 mg, PO)	Healthy male volunteers (*n* = 15)	No significance differences in systemic exposure	[60]
CYP3A4 inhibition	Ritonavir (200 mg, PO, BID, 4 or 14 days)	Prednisolone (20 mg, PO)	Healthy volunteers (*n* = 10)	1.4- and 1.3-fold increase in AUC_inf_ after administration of ritonavir for 4 or 14 days	[58]
	Diltiazem (180 mg, PO, BID, 3 days)	Prednisolone (15 mg, PO)	Healthy volunteers (*n* = 8)	1.2- and 1.08-fold increase in AUC and C_max_	[59]
	Itraconazole (400 mg, PO for 1 day and then 200 mg, PO for 3 days)	Prednisolone (60 mg, PO)	Healthy male volunteers (*n* = 14)	No significance differences in systemic exposure	[61]
	Itraconazole (200 mg, PO, 4 days)	Methylprednisolone (16 mg, IV)	Healthy volunteers (*n* = 9)	2.6-fold increase in AUC_inf_	[62]
	Itraconazole (400 mg, PO for 1 day and then 200 mg, PO for 3 days)	Methylprednisolone (48 mg, PO)	Healthy male volunteers (*n* = 14)	2.5- and 1.6-fold increase AUC_24h_ and C_max_	[61]
	Itraconazole (200 mg, PO, 4 days)	Methylprednisolone (16 mg, PO)	Healthy volunteers (*n* = 10)	3.9- and 1.9-fold increase AUC_24h_ and C_max_	[63]
	Grapefruit juice (200 mL, double-strength, PO, TID, 2 days and then 0.5 h and 1.5 h after methylprednisolone administration)	Methylprednisolone (16 mg, PO)	Healthy volunteers (*n* = 10)	1.7- and 1.3-fold increase in AUC_inf_ and C_max_	[64]
	Diltiazem (180 mg, PO, 4 days)	Methylprednisolone (0.3 mg/kg, IV)	Healthy male volunteers (*n* = 5)	1.5-fold increase in AUC	[65]
	Aprepitant (125 mg, PO on day 1, and 80 mg, PO on day 2/3)	Methylprednisolone (120 mg, IV on day 1, and 40 mg, PO on day 2/3)	Healthy volunteers (*n* = 10)	1.3- and 2.5-fold increase in AUC_24h_ at day 1 and 3	[66]
	Nefazodone (100 mg for 3 days, 150 mg for 2 days, and 200 mg for 5 days, BID, PO)	Methylprednisolone (0.6 mg/kg, IV)	Healthy volunteers (*n* = 8)	2.2-fold increase in AUC	[67]
	Ketoconazole (200 mg, PO, 4 days)	Budesonide (3 mg, PO)	Healthy male volunteers (*n* = 8)	6.5-fold increase in AUC_24h_	[68]
	Grapefruit juice (200 mL, regular strength, PO, TID, 4 days)	Budesonide (3 mg, PO)	Healthy male volunteers (*n* = 8)	1.7-fold increase in AUC and C_max_	[69]
